# The fungal endophyte *Epichloë typhina* improves photosynthesis efficiency of its host orchard grass (*Dactylis glomerata*)

**DOI:** 10.1007/s00425-015-2337-x

**Published:** 2015-06-10

**Authors:** Piotr Rozpądek, K. Wężowicz, M. Nosek, R. Ważny, K. Tokarz, M. Lembicz, Z. Miszalski, K. Turnau

**Affiliations:** Institute of Environmental Sciences, Jagiellonian University, Gronostajowa 7, 30-387 Kraków, Poland; Institute of Plant Physiology, Polish Academy of Sciences, Niezapominajek 21, 30-239 Kraków, Poland; Institute of Biology, Pedagogical University, Podbrzezie 3, 31-054 Kraków, Poland; Malopolska Centre of Biotechnology, Jagiellonian University, Gronostajowa 7a, 30-387 Kraków, Poland; Institute of Plant Biology and Biotechnology, University of Agriculture, 29 Listopada 54, 31-425 Krakow, Poland; Department of Plant Taxonomy, A. Mickiewicz University, Umultowska 89, 61-614 Poznań, Poland

**Keywords:** Endophyte, *Dactylis glomerata*, *Epichloë typhina*, Photosynthesis, NPQ

## Abstract

**According to the results presented in this paper the fungal endophyte*Epichloë typhina*significantly improves the growth, PSII photochemistry and C assimilation efficiency of its host*Dactylis glomerata***.

In this paper, we present a comprehensive study of the impact of the endophytic fungi *Epichloë typhina* on its plant hosts’ photosynthesis apparatus. Chlorophyll *a* fluorescence, gas exchange, immuno-blotting and spectrophotometric measurements were employed to assess photosynthetic performance, changes in pigment content and mechanisms associated with light harvesting, carbon assimilation and energy distribution in *Dactylis glomerata* colonized with *Epichloë typhina*. According to the results presented in this study, colonization of *D. glomerata* results in improved photosynthesis efficiency. Additionally, we propose a new mechanism allowing plants to cope with the withdrawal of a significant fraction of its energy resources by the endophytic fungi. The abundance of LHCI, LHCII proteins as well as chlorophyll *b* was significantly higher in E+ plants. Malate export out of the chloroplast was shown to be increased in colonized plants. To our knowledge, we are the first to report this phenomenon. *Epichloë* colonization improved PSII photochemistry and C assimilation efficiency. Elevated energy demands of E+ *D. glomerata* plants are met by increasing the rate of carbon assimilation and PSII photochemistry.

**Electronic supplementary material:**

The online version of this article (doi:10.1007/s00425-015-2337-x) contains supplementary material, which is available to authorized users.

## Introduction

Nearly all plants associate in symbiotic relationships with endophytic fungi. Since the early 90s these interactions have been the subject of intensive research, allowing us to gain a general understanding of the role of fungal symbionts in plant physiology and ecology (Schardl 2001; Rodriguez et al. 2009). Many issues, however, evoke serious controversy. Questions concerning endophyte-imposed benefits on plant growth are still being raised. According to some authors endophytic fungi colonization is beneficial for its plant host (Stein et al. 2008; Oelmüller et al. 2009). On the contrary, others classify this interaction as “mild” biotrophic parasitism (Faeth and Sullivan 2003; Saikkonen et al. 2004). The association of plants with symbiotic fungi has been proven to significantly affect plant growth and development. Out of the numerous aspects of plant physiology affected by symbiotic fungi, growth (biomass production), resistance against pathogens and herbivores, nutrient uptake and plant reproduction figure to have the biggest impact on agriculture and shaping of plant communities (Kannadan and Rudgers 2008; Rudgers and Swafford 2009; Yuan et al. 2009; Hartley and Gange 2009; Johnson et al. 2013). Given the potential for biotechnology, there is great interest in understanding the mechanisms of this interaction (Aly et al. 2010; Johnson et al. 2013).

*Dactylis glomerata*, a C_3_ grass species, spread over the northern hemisphere has been shown to form a symbiotic association with the fungal endophyte *Epichloë typhina*, a member of the Clavicipitaceae family. *Epichloë* fungi are known for their ability to synthesize bioactive compounds, serving as plant protective agents against pathogens and herbivores (Yuan et al. 2009; Czarnoleski et al. 2012). This, however, has been shown to severely affect the health of livestock and the quality of dairy products. Milk from areas where the fungus has spread, due to its toxicity, has been banned from local markets (Bernard et al. 1993; Schmidt and Osborn 1993; Young et al. 2013). This phenomenon is widely known in the USA, New Zealand and Australia but recently it has been gaining significance in Europe. In Central Europe *Epichloë* endophytes have been reported in several grass species including *Festuca*, *Lolium*, *Puccinellia*, *Agropyron*, *Poa, Elymus* and others (Christensen et al. 2002; Górzynska et al. 2011; Lembicz et al. 2013).

*E. typhina,* similar to other members of *Epichloë* resides within host shoot tissues, being almost completely confined to the apoplast. During its sexual reproduction it forms characteristic, visible structures—the stromata—causing “choke disease” limiting seed formation, but supporting clonal growth (Schardl 2001). According to previous reports fungi from the *Epichloë* genus stimulate the growth of its host plant during certain periods of plant ontogenesis (Olejniczak and Lembicz 2007). The fungal partner benefits from the symbiosis withdrawing assimilated photosynthetic carbon and additional elements, particularly nitrogen and sulfur, in sufficient abundance for protein and other essential metabolite synthesis (Christensen et al. 2002).

One way to support elevated energy demands is to improve carbon assimilation efficiency. Photosynthesis requires the orchestrated action of light-driven energy production (ATP and NADPH) and CO_2_ assimilation in the Calvin–Benson–Bassham cycle. Alterations in the equilibrium between ATP and NADPH production and carbon assimilation due to increased energy demands or stress may substantially alter photosynthetic performance. Chloroplasts have evolved significant energy flexibility mechanisms allowing them to cope with environmental challenges. This plasticity derives from the ability of the photosynthetic apparatus to distribute absorbed energy quantum in a very flexible manner. Out of the many routes of energy management the cyclic electron flow, non-photochemical quenching mechanism (NPQ), malate valve and the water–water cycle seem to be the most important (Kramer and Evans 2011).

Non-photochemical quenching (heat dissipation of excess energy) is performed by at least three distinct mechanisms: energy-dependent quenching (qE), photoinhibitory quenching (qI) and state transition quenching (qT). In non-stressed leaves under moderate to saturating light conditions qE is the major component of NPQ (Baker 2008). It is activated upon light-driven, reversible acidification of the thylakoid lumen and activation of the xanthophyll cycle.

Thylakoid lumen acidification and its redox state is controlled by the malate valve and the malate dehydrogenase (MDH) in particular. It catalyzes the reduction of oxaloacetate to malate and exports the latter out of the chloroplast (the malate valve) enabling de-acidification of the thylakoid lumen (Fridlyand et al. 1998). It has been suggested to play a role in maintaining an appropriate ATP/NADPH ratio, allowing energy production, distribution and carbon assimilation during stress and acclimation. It also de-acidifies the thylakoid lumen by exporting H^+^ in the form of malate to the cytosol (Scheibe 2004).

The main source of carbon for green plants is photosynthesis, thus we hypothesized that *D. glomerata* infected with *E. typhina* must have developed photosynthesis-related mechanisms allowing it to meet elevated energy demands. According to previous reports, CO_2_ fixation efficiency is decreased in *Lolium perenne* by *E. typhina’s* asexual state-*Neotyphodium lolii*. It does not, however, affect photochemistry (Spiering et al. 2006). Additionally, Amalric et al. (1999) and Marks and Clay (1996) have shown that in high temperature E+ (endophyte) *N. lolii* positively affects photosynthesis in perennial ryegrass. Improved water use efficiency and other physiological traits observed in E+ plants suggest the possibility that fungal endophytes may indeed positively influence plant photosynthesis (Arachevaleta et al. 1989; Richardson et al. 1993). Previously performed pilot experiments on *D. glomerata* in its natural environment confirmed this assumption. Photosynthesis efficiency as well as the content of photosynthetically active pigments was increased upon *E. typhina* colonization. These results led us to examine the phenomenon of endophyte-induced photosynthesis efficiency in a more detailed fashion.

In this study, we attempted to assess the impact of the endophytic *E. typhina* on the photosynthesis apparatus of orchard grass.

## Methods

### Plant material

*D. glomerata* L. plants with and without developed stromata were obtained from the campus of University of Poznań (Poland) in May 2012. Plant rhizomes of uniform size (18 rhizomes from plants with stromata and 18 rhizomes from plants without visible stromata) were collected from the mother grass, planted in plexiglass rhizoboxes (40 × 20 × 4 cm) and grown from May until November 2012 in the substratum from the campus of the Jagiellonian University Kraków, Poland in the University garden (natural, field conditions). During harvest and measurements a *c.a*. 10 h photoperiod and 150 µmol m^−2^ s^−1^ PAR were present. Plants were irrigated on daily basis. No fertilizers or pest control was used during vegetation. At the end of the experiment, a uniform number of leaves from two plants (for one sample) were collected at the beginning of the photoperiod (0700 h) and frozen in liquid nitrogen. Nine samples for each treatment (E+ and control) were collected.

### Fungi identification

The fungus was identified using the nucleotide sequences of the *β*-tubulin gene (*tubB*). DNA isolation, amplification and sequencing were performed according to the procedure described previously by Brem and Leuchtmann (2003) and Lembicz et al. (2010). A fragment that contained the first three introns of the *β*-tubulin gene (*tubB*) was amplified using primers by Craven et al. (2001). Sequences were compared to reference (Lembicz et al. 2011) and deposited in GenBank (National Center for Biotechnology Information, Bethesda, Maryland, USA) under accession number HM007554-HM007560.

Before measurements and harvest (plant material collection) intercellular mycelia were observed using a stereomicroscope, and the presence of the fungus as an endophyte in vegetative tissues of *D. glomerata* was confirmed. Isolates were obtained from surface-sterilized tissues of stroma that formed tillers grown in axenic cultures.

The presence of the endophyte in *D. glomerata* tissues was monitored by microscope observation three times during vegetation—at the beginning of the experiment (May), in the middle (August) and at the end of the experiment, before harvest (November). Only plants with confirmed presence/absence of the endophyte in *D. glomerata* tissues were examined.

### Chlorophyll content determination

Chlorophyll content was determined according to the method described by Barnes et al. (1992). Chlorophyll *a* and *b* absorbencies were measured at 665 and 648 nm with the CECIL 9500 spectrophotometer and calculated for chlorophyll concentration in fresh weight in nine replicates.

### SDS-PAGE and immuno-blotting

Plant leaves were frozen in liquid nitrogen, grounded with a mortar and pestle, and homogenized in ice-cold 100 mM Tricine–Tris buffer pH 8.0, containing 100 mM MgSO_4_, 1 mM DTT and 3 mM EDTA (Miszalski et al. 1998), flash frozen in liquid nitrogen and sonicated for 20 s. Repeated freezing and sonication was performed in three series (to a point where the supernatant was greener than the pellet) according to the liquid nitrogen + soni–freeze–thaw method described in Western blotting–tips and trouble-shooting (http://www.agrisera.com/cgi-bin/ibutik/AIR_ibutik.pl?funk=Webbsida&ID=152). After extraction, probes were centrifuged for 10 min at 10,000*g* at 4 °C. Soluble protein content was quantified according to Bradford (1976) using BSA as a standard. For protein denaturation, samples were treated with a 50 mM Tris containing 10 M urea, 2 % β-mercaptoethanol and 4 % SDS and heated at 70 °C for 10 min. Electrophoresis was carried out in a Mini-PROTEAN tetra System (BioRad, USA) in polyacrylamide (PAA) gels containing 4 % SDS according to Laemmli (1970). Protein separation for Rubisco LSU (large subunit) quantification was performed in 4 % stacking (pH 6.8) and 10 % resolving (pH 8.8), 5 µg of total protein was loaded per lane. For the remaining proteins 5.8 % stacking (pH 6.8) and 12 % resolving (pH 8.8) gels were used. 10 µg for D1, Lhca2, Lhca3 and PsaC of total protein was loaded per lane. Electrophoresis was carried out at constant 24 mA for the first 15 min (in the stacking gel), followed by 36 mA until full separation of the protein marker ladder (Thermoscientific, LT). Following electrophoresis, transfer to PVDF membranes (semi-dry) was performed using TransBlot Turbo Transfer System (BioRad, USA). For immunodetection Agrisera (S) primary polyclonal antibodies in the following dilutions were used: Rubisco LSU: 10,000, D1: 1:10,000, PsaC: 1:1,000, Lhca2: 1:2000, Lhcb3: 1:2000. After an overnight incubation period at 4 °C, membranes were treated with secondary antibodies conjugated with alkaline phosphatase (Sigma, USA) for 1.5 h (1:5000 dilution). Specific proteins were visualized by soaking membranes in 20 % BCIP/NBT solution for 1 min. After drying, membranes were scanned with an office scanner. Analysis was performed in triplicates. Densitometric analysis was performed with the Image J software (NIH, USA).

### NADPH-malate dehydrogenase activity

The activity of NADPH-malate dehydrogenase (NADPH-MDH, EC 1.1.1.37) was determined in frozen leaf tissue (0.4 g) homogenized on ice in 1.0 ml of 0.1 M Hepes–NaOH pH 8.0, containing 5 mM DTT, 0.5 % (w/v) bovine serum albumin and polyvinylpyrrolidone 40 (0.5 % w/v). The homogenate was centrifuged at 10,000*g* for 5 min (4 °C). The activity measurement was performed according to Holtum and Winter (1982). Analysis was performed in nine replicates.

### Chlorophyll *a* fluorescence measurements

PSII photochemistry was analyzed with the Dual-PAM (Walz, D) fluorometer. Light curves were recorded in 0–851 µmol s^−1^ m^−2^ light range, with ten 1-min illumination steps on light-acclimated leaves. Light curves were collected from seven plants from each treatment at the end of the experiment.

The electron transport rate was calculated according to the following equations:

$${\text{ETR}} = {\text{Y(II)}}\cdot{\text{PAR}}\cdot0. 8 4\cdot0. 5$$

where PAR is the photosynthetic photon flux density (μmol photons m^−2^ s^−1^), 0.84 is the theoretical absorption factor of green leaves and 0.5 expresses the equal distribution of excitation energy between PSII and PSI (Maxwell and Johnsons 2000); the complementary quantum yields of energy conversion in PSII were calculated using the Dual-PAM software according to equations by Kramer et al. (2004):

$${\text{Y(II)}} = (F^{\prime}_{\text{m}} - F)/F^{\prime}_{\text{m}}$$

$${\text{Y(NPQ)}} = 1- {\text{Y(II)}} - 1/({\text{NPQ}} + 1+ {\text{qL(}}F_{\text{m}} /F_{0} - 1 ))$$

$${\text{Y(NO)}} = 1/({\text{NPQ}} + 1+ {\text{qL}}\; (F_{\text{m}} /F_{0} - 1 ))$$

$${\text{qL}} = (F^{\prime}_{\text{m}} - {\text{F}})/(F^{\prime}_{\text{m}} - F^{\prime}_{ 0} )\;F^{\prime}_{ 0} /F$$

where *F*_0_′ is not measured, it is approximated according to Oxborough and Baker (1997):

$$F^{\prime}_{ 0} = F_{0} /(F_{\text{v}} /F_{\text{m}} + F_{0} /F^{\prime}_{\text{m}} )$$

NPQ measurements (induction curves) were performed on dark-acclimated leaves at actinic light intensities ranging from 37 to 126 µmol s^−1^ m^−2^. Actinic light intensities were selected according to light conditions directly before the measurement. The induction curve was performed according to the manufacturers’ instruction, with modifications. The actinic light phase of the measurement was prolonged to 10 min, with a 20-s relaxation period after application of each saturating pulse. NPQ measurements were performed on nine plants from each treatment at the end of the experiment according to the following equation:

$${\text{NPQ}} = (F_{\text{m}} - F^{\prime}_{\text{m}} )/F^{\prime}_{\text{m}}$$

### Gas exchange, photosynthesis efficiency measurements

Gas exchange measurements including stomatal conductance were carried out on *D. glomerata* leaves with a portable, open gas exchange system (Li-6400, Li-Cor, Lincoln NE) equipped with a 6400-02B LED Light Source in a 6 cm^2^ cuvette. Measurements were performed in CO_2_ saturated conditions (650 µmol mol^−1^); 500 µmol s^−1^ of air flow, 40–45 % relative humidity, 25 °C leaf temperature and under the 400 µmol (quantum) m^−2^ s^−1^ red light intensity.

The *A*/Ci (assimilation at given intracellular CO_2_ concentration) response curves were registered in 400 µmol (quantum) m^−2^ s^−1^ of light and in the 0–1200 µmol CO_2_ m^−2^ s^−1^ range. The CO_2_ compensation points were calculated by extrapolation of the logarithmic function equations: *y* = 5.4916ln (*x*) − 23.773 (endophyte) and *y* = 5.3657ln (*x*) − 25.422 (control) based on the CO_2_ response curves data obtained from measurements. Gas exchange measurements were performed in five replicates.

## Results

### Plant biomass

**Fig. 1:**
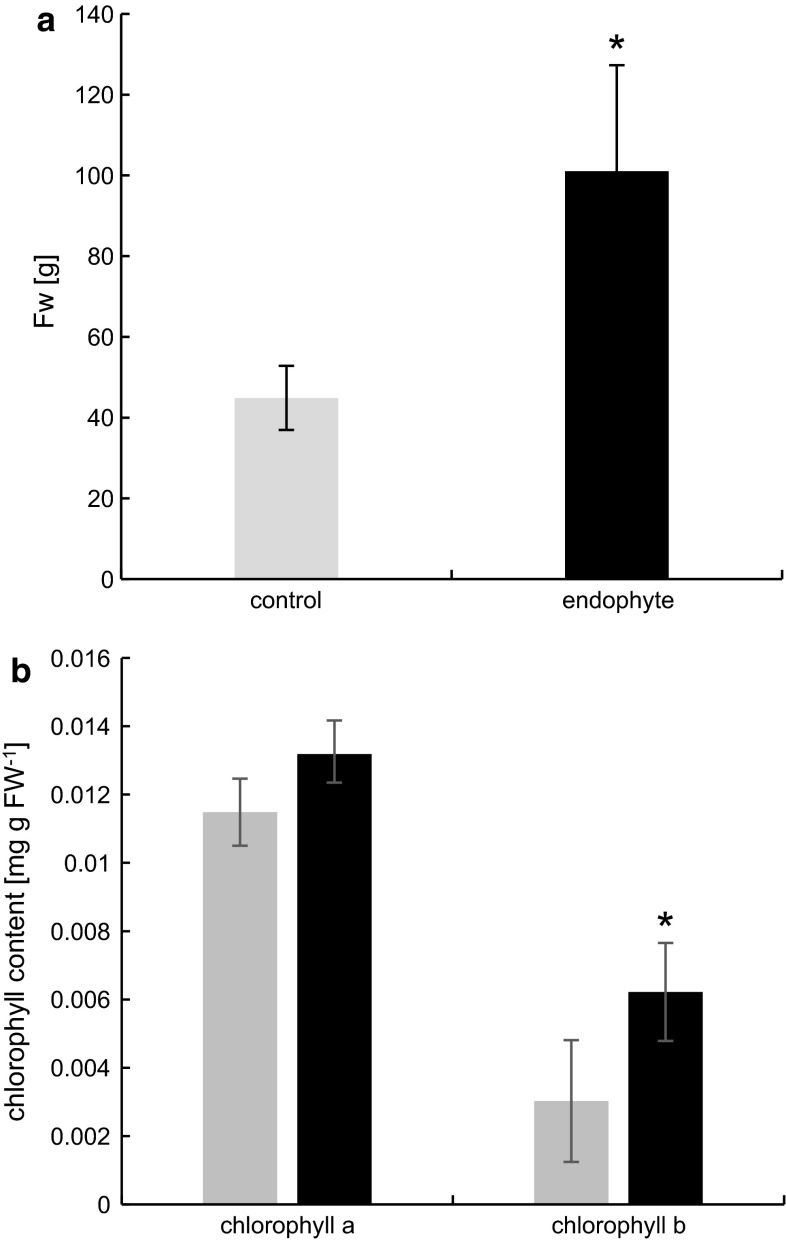
Fresh weight of E+ and E− *D. glomerata* (**a**). *Marks* represent mean values from 18 measurements (*n* = 18 ± SD). Chlorophyll *a* and *b* concentration of E+ and E− *D. glomerata* (**b**). *Marks* represent mean values from nine independent measurements (*n* = 9 ± SD). Stars above symbols indicate statistically significant differences in relation to control at *P* ≤ 0.05

*D. glomerata* plants grown from rhizomes yielded significantly higher biomass (an over twofold difference) compared to their uninfected counterparts (Fig. 1a), but did not produce seeds.

### Fluorescence measurements

**Fig. 2:**
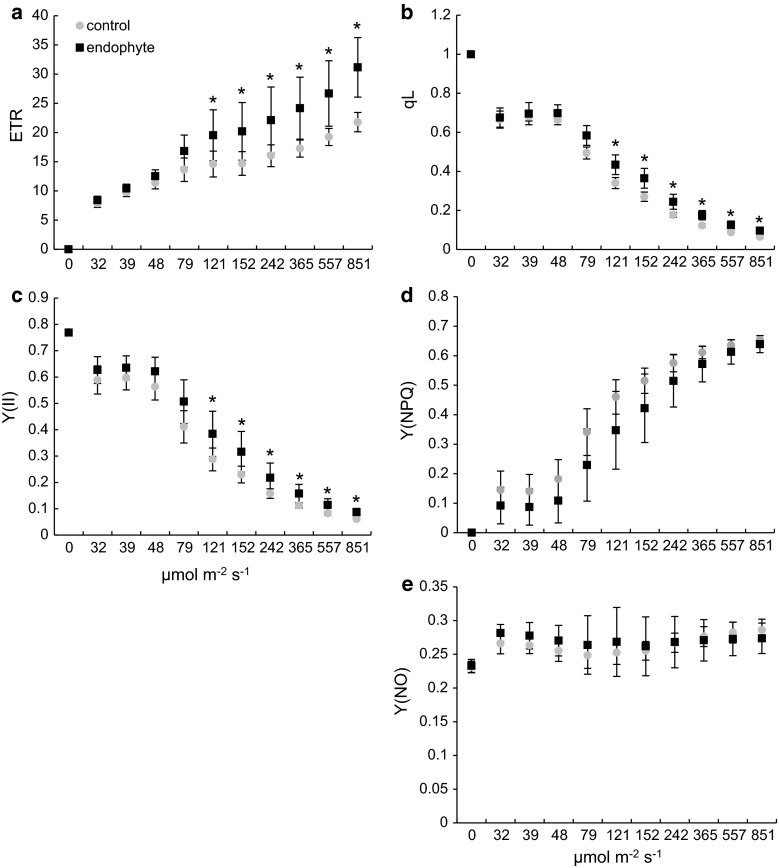
Photosystem II efficiency measurements of *D. glomerata* colonized with *E. typhina*—*black squares* and control—*grey circles*. (**a**) Electron transport rate (ETR), (**b**) photochemical coefficient qL, (**c**) the effective quantum yield of PSII Y(II), (**d**) regulated energy dissipation Y(NPQ) and (**e**) non-regulated energy dissipation Y(NO). *Light curves* were registered from light-acclimated plants. *Marks* represent mean values from seven independent measurements (*n* = 7 ± SD). *Stars* above *symbols* indicate statistically significant differences in relation to control at *P* ≤ 0.05

The electron transfer rate (ETR) a relative measure of the rate of electron flow through PSII (rate of charge separation at PS II reaction centers) (Fig. 2a) and qL (Fig. 2b) illustrating the fraction of open PSII reaction centers was increased in E+ plants in a similar fashion. A 43 % increase at 121 µmol s^−1^ m^−2^ was shown, reaching 69 % at 851 µmol s^−1^ m^−2^ for the former and from 18 to 50 % for the latter. From light curve analysis it seems clear that the presence of the endophyte improves the plants ability to convert incident light into chemical energy in a light-dependent manner. At given light conditions the efficiency of PTEC of E+ plants increases proportionally to light intensity. The effective quantum yield of PSII (Y(II)), illustrating the fraction of the absorbed quanta converted into chemically fixed energy was improved in *Epichloë*-colonized plants in PAR range from 121 to 851 µmol s^−1^ m^−2^ (Fig. 2c). In light intensities ranging from 0 to 79 µmol s^−1^ m^−2^ no differences were recorded, probably due to limited saturation of the photosynthetic apparatus, insufficient to utilize its full light harvesting potential. The effective quantum yield of E+ compared to E− plants increased by 23–43 % with growing light intensities (Fig. 1c). During light curve recording, the yield of non-photochemical quenching Y(NPQ) and the non-regulated energy dissipation Y(NO) (Fig. 2d, e) was not influenced by the presence of *Epichloë* endophytes in *D. glomerata* tissues at most tested light intensities. At low PAR (32–79 µmol s^−1^ m^−2^) statistically significant decreases were observed (data not shown). This ambiguity leads us to perform a more precise NPQ determination after dark acclimation (induction curve).

At the onset of photosynthesis, i.e., after dark acclimation electrons from PSI cannot be transferred to the Calvin cycle because its enzymatic machinery is inactive and its activation occurs at lower rates than photochemistry (Cardol et al. 2010). This allows to study electron flux through PSII, in a more detailed fashion.

NPQ determinations in dark-acclimated plants showed that endophyte colonization resulted in alterations in NPQ activation kinetics (Fig. 4a). Typically after dark acclimation a rapid induction of NPQ occurs, followed by a gradual decline before reaching steady state. The rapid activation of NPQ in control plants can be attributed to an instantaneous buildup of a proton gradient across the thylakoid membrane and NPQ activation (a typical response). However, in plants colonized by *Epichloë* endophytes NPQ activation kinetics significantly differed from that found in control. NPQ gradually rose, to reach its steady state after 12 light pulses (approximately 5 min from the onset of actinic light). The activation of electron sinks, the Calvin cycle in particular resulted in a gradual decline. Steady-state NPQ did not vary in the presence of the endophyte, however, NPQ activation differed from control significantly. After eight excitation pulses NPQ in *Epichloë*-colonized plants reached control values. No rapid NPQ activation and gradual decline was observed. Non-photochemical quenching rose to a certain level to reach steady-state values.

Light intensity plays a pivotal role in NPQ activation, thus to determine whether NPQ activation kinetics in *D. glomerata* will change at higher light intensities we performed additional experiments at higher actinic light, with stronger saturation pulses. The rationale here was to verify whether lower NPQ at the initial stage of activation is a result of a higher electron flow capacity, allowing electron flow at fairly low actinic light preventing thylakoid lumen acidification and NPQ activation. In 126 µmol s^−1^ m^−2^ (the highest tested) of actinic light we observed a similar, but less significant reaction of the photosynthetic apparatus suggesting the presence of other mechanisms sustaining electron flow through PSII (data not shown).

### Gas exchange measurements

**Fig. 3:**
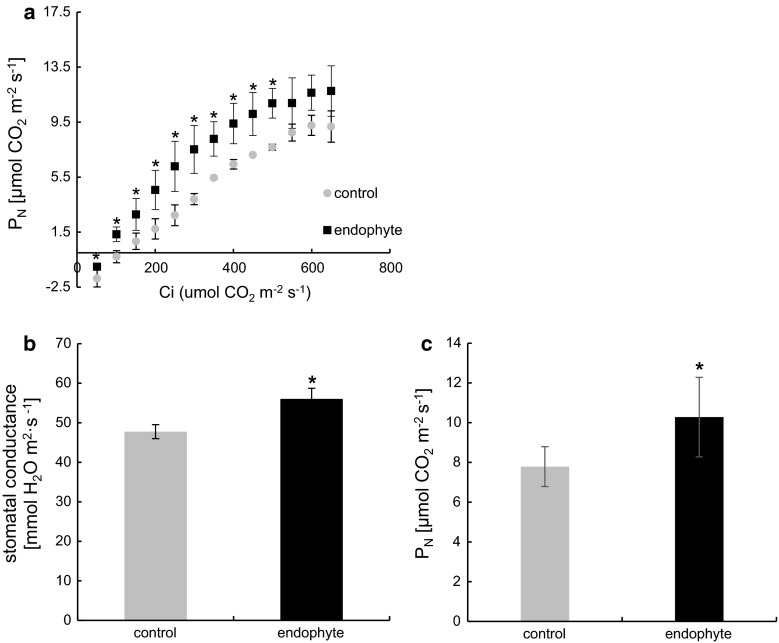
*A*/Ci curve (**a**) and stomatal conductance (**b**), and nett photosynthesis (**c**) at optimal CO_2_ levels of *D. glomerata* colonized with *E. typhina*—*black squares* and control—*grey circles*. *Marks* represent mean values from five independent measurements (*n* = 5 ± SD). *Stars* above *symbols* indicate statistically significant differences in relation to control at *P* ≤ 0.05

To verify fluorescence analysis in the context of carbon assimilation we performed gas exchange measurements according to which *E. typhinë* improves nett photosynthesis at saturated CO_2_ (650 µmol mol^−1^) concentrations (Fig. 3c) in *D. glomerata*. Nett photosynthesis averaged 10.3 µmol CO_2_ m^−2^ s^−1^ in E+ plants and 7.8 µmol CO_2_ m^−2^ s^−1^ in control.

To assess the efficiency of carbon assimilation in the biochemical phase of photosynthesis (Calvin cycle) we performed the *A/*Ci curve and found that the rate of carboxylation in E+ plants was significantly higher in CO_2_ concentrations up to 500 µmol m^−2^ s^−1^. At higher CO_2_ differences in the rate of carbon assimilation were insignificant (Fig. 3a). The CO_2_ compensation point as shown in Fig. 3a was significantly lower (close to twofold) in our model, suggesting a more effective rate of CO_2_ carboxylation in E+ plants.

Stomatal conductance (Fig. 3b) was significantly increased (by 15 %) in E+ *D. glomerata*, but its transpiration rate, as well as water use efficiency was similar to that found in control (data not shown).

### Chlorophyll concentration

The concentration of total chlorophyll was significantly improved in *Epicholë*-colonized *D. glomerata*, by approximately 33 %. In E+ 0.0194 mg g FW^−1^, whereas in control 0.0145 mg g FW^−1^ was recorded. No statistically significant differences were reported in the concentration of chl *a*, however, a twofold increase in the concentration of chl *b* was shown in (Fig. 1b). In E+ 0.0062 mg g FW^−1^, whereas in control 0.003 mg g FW^−1^ was recorded. The chl *a*/*b* ratio was also changed in infected plants from 4:1 to 2:1.

### Immuno-blot analysis of chloroplast proteins

**Fig. 4:**
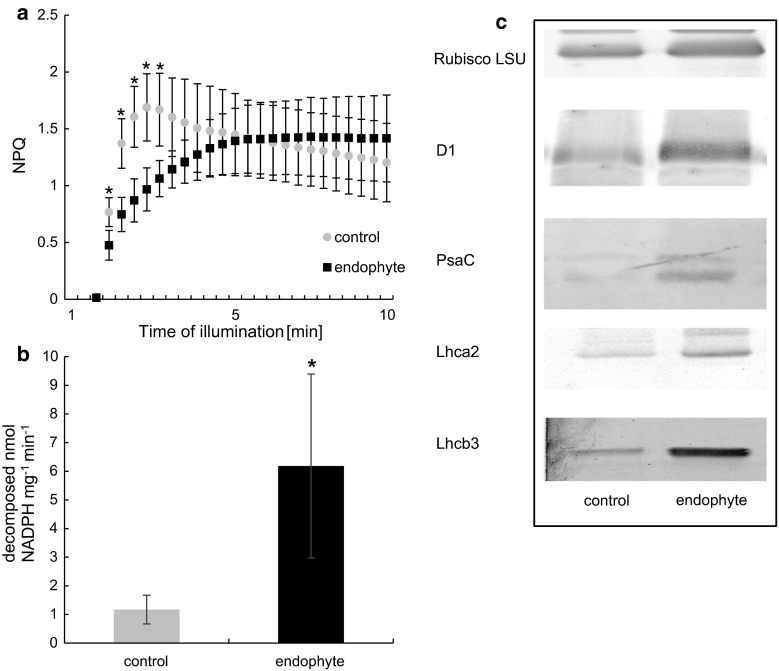
NPQ of *D. glomerata* colonized with *E. typhina*—*black squares* and control—*grey circles*. Induction curves were registered after dark acclimation at 63 µmol s^−1^ m^−2^ actinic light intensity (**a**). NADPH-MDH activity (**b**) from *D. glomerata* colonized with *E. typhina*—*black bars* and control—*grey bars*. *Marks* mean values from nine independent measurements (*n* = 9 ± SD). *Stars* above *symbols* indicate statistically significant differences in relation to control at *P* ≤ 0.05. Typical immunoblots of SDS-PAGE-separated polypeptides (**c**) of Rubisco (5 µg of total protein per lane), D1 (10 µg of total protein per lane), PsaC (10 µg of total protein per lane) and Lhca2 (10 µg of total protein per lane) from *D. glomerata* colonized with *E. typhina* and control

To determine potential changes in photosystem antennae size/abundance, the content of selected PSII and PSI proteins was assessed. A twofold increase in the abundance of the PSII protein D1 and over threefold increase in the abundance of Lhcb3 chl *a*/*b*-binding proteins was shown. The content of PSI proteins: PsaC and Lhca2 chl *a*/*b*-binding proteins has been shown to be significantly (by over twofold) increased in *Epichloë*-colonized *D.glomerata* (Fig. 4c; Table 1).

**Table 1:** Relative abundance of PSI and PSII proteins in *D. glomerata* leaves inoculated with *E. typhina* and control

Protein	Relative abundance	
	Control	Endophyte
Rubisco LSU	39,237 ± 5585a	48,939 ± 3995b
D1	24,952 ± 23a	48,189 ± 2167b
Psa C	2582 ± 217a	7280 ± 750b
Lhca 2	10,812 ± 347a	23,573 ± 1993b
Lhca 3	7432 ± 805a	26,273 ± 8687b

PSI and PSII proteins from crude extracts were separated in SDS-PAGE and transferred to PVDF membranes. Densitometric analysis of immunoblots was performed with ImageJ (NIH, US). Different letters indicate statistically significant differences at *P* ≤ 0.05. All analysis were performed in triplicates

### NADPH-MDH activity

The activity of the NADPH-MDH enzyme was significantly (sixfold) increased in infected *D. glomerata.* In E+ plants 6.183 nmol min^−1^ mg^−1^ protein of NADPH was decomposed by NADPH-MDH compared to 1.17 nmol min^−1^ mg^−1^ protein in control (Fig. 4b).

## Discussion

In previous studies, selected strains of *E. typhinë* delivered benefits in productivity and persistence of *Lolium perenne* (Popay et al. 1999; Hume et al. 2007, 2009). The improved performance of these endophyte-infected grasses has been attributed to their effects on insect herbivores (Johnson et al. 2013). In this study, a significant yield improvement of *D. glomerata* colonized with commonly occurring in Central Europe strain of *E. typhinë* grown in semi-controlled conditions was shown. According to Easton (2007) the effect of *Epichloe* endophytes on grass species is small and inconsistent under controlled conditions.

Since no coherent data explaining the phenomenon of *E. typhina*-induced grass growth improvement have been presented yet, we propose that the presence of the beneficial fungi shifts plant metabolism from defense to growth allowing the plant to utilize available resources for photosynthesis and carbon assimilation. This allows the plant to “feed” its fungal partner and yield high biomass. Numerous studies with mycorrhizal plants have shown increased stress resistance and a significant improvement in photosynthesis efficiency upon fungal colonization (Aloui et al. 2011; Ruiz-Lozano et al. 2012).

Here, the efficiency of electron transport through PSII was shown to be improved in E+ *D. glomerata* plants, suggesting a higher rate of solar energy utilized for photochemistry. Increased electron density flux indicates higher ATP and NADPH production in the PETC (photosynthetic electron transport chain), employed for carbon assimilation in the Calvin cycle. Improved CO_2_ assimilation measured by gas exchange seems to support this point of view.

Besides photochemical reactions, incident light energy absorbed by the photosynthetic apparatus can be dissipated as heat or cause potential damage by generation of deleterious ROS (reactive oxygen species) when in excess (Moller and Sweetlove 2010). At high PAR intensities the amount of absorbed quanta dissipated as heat, Y(NPQ) and potentially harmful (Y(NO) non-regulated energy dissipation) increase with a parallel decrease in Y(II). In this study, no significant changes in Y(NPQ) and Y(NO) were shown during light curve recording. Y(NO) values were fairly low and steady, independent of irradiance, suggesting that the majority of the incident light energy is either converted into ATP and NADPH or dissipated as heat.

Photosynthesis is limited by biophysical and biochemical barriers such as stomatal conductance and carboxylation capacity. Carbon assimilation is strongly related to CO_2_ accessibility, which in turn depends on the intercellular carbon dioxide concentration and limitations in its diffusion. Stomatal conductance impacts gas diffusion rates resulting in a decline of chloroplastic CO_2_ concentration and subsequently limits photosynthesis (Sharkey et al. 2007; Centritto et al. 2009). Even though stomatal conductance in colonized *D. glomerata* plants was shown to be significantly higher no differences in transpiration rate were reported. These results are difficult to interpret in the context of gas exchange; however, the decreased CO_2_ compensation point of E+ plants suggests that the internal concentration of CO_2_ may had been higher, improving nett photosynthesis. Higher internal CO_2_ concentrations indicate an increased rate of carbon assimilation by Rubisco. Increased carboxylation may derive from increased Rubisco activity or abundance.

Improved electron flow rate and carbon assimilation should be accompanied by improved light harvesting. Total chlorophyll concentration was significantly increased in E+ plants. Measurements of chl *a* and chl *b* revealed that the amount of chl *b* (over 100 % increase) accounted for the observed difference. Chlorophyll *b* transfers excitation energy to chlorophylls from reaction centers and is necessary for proper assembly and stabilization in thylakoid membranes and for appropriate functioning of most LHC complexes (Hoober and Eggink 2001). Reduced chl *a*/*b* ratio characteristic for plants grown in low-light conditions usually results in increased grana stacking that is often associated with modifications in protein content. Tobacco plants overexpressing the *CAO* gene (chlorophyllide *a* oxygenase) responsible for chl *b* synthesis have been shown to have improved light harvesting capacities, increased electron transport and carbon dioxide fixation independently of light conditions (Biswal et al. 2012). The improved PS(II) efficiency and increased chl *b* content–characteristic for PSII—led us to speculate that in *Epichloë*-colonized plants the abundance of PSII should be elevated. To verify this hypothesis, we quantified the abundance of the PSII proteins PsbA and Lhcb3. PsbA along with several other proteins (D2, the α- and β-subunits of cytochrome b559, and the PsbI protein) form the PSII reaction center complex. The D1/D2 heterodimer binds the manganese cluster involved in water oxidation in PSII (Nanba and Satoh 1987, Sun et al. 2007). Lhcb3 is a preferential site of regulation of the antenna function in excess light conditions (Caffarri et al. 2004). A pronounced increase in the abundance of these proteins was shown in colonized plants, indicating a higher density of PSII in E+ plants.

According to our results, an increase of PSI-associated proteins was present in E+ plants. Lhca2 is one of four PSI Lhca proteins. It is associated with PsaA and to PsaJ via gap chlorophylls (Schmid 2008). PsaC is a conserved, Fe–S-binding protein present in all known PSI complexes. It is tightly bound to the PsaA/PsaB heterodimer and coordinates the Fe–S clusters FA and FB through two cysteine-rich domains. PsaC has been shown to be essential for in vivo assembly of PSI in *Chlamydomonas reinhardtii* (Takahashi et al. 1991, 1992). Taken together, we can conclude that improved photosynthetic performance in endophyte-colonized *D.glomerata* was due to improved harvesting capacities.

The photosynthesis apparatus of *Populus alba* and *Medicago truncatula* inoculated with AMF (arbuscular mycorrhiza fungi) species from the *Glomus* genus behaved similarly. According to proteomic and gene expression studies, the expression of Rubisco, several chlorophyll-binding proteins and PSII and I structural proteins was increased in AMF+ plants. The efficiency of carbon assimilation and PSII photochemistry was also improved in a manner similar to that found in E+ *D. glomerata* (Aloui et al. 2011; Cicatelli et al. 2010, 2012; Lingua et al. 2008). Even though AMF and fungal endophytes belong to distinct groups of fungi, both can be beneficial for their host. As shown in this study beneficial symbionts may similarly affect the photosynthesis apparatus of their host resulting in improved growth and/or stress resistance.

Significant changes in NPQ activation kinetics in E+ plants were recorded. Since carbon assimilation cannot serve as a sink for electrons at the onset of actinic light phase of the NPQ measurement, a mechanism changing NPQ activation kinetics, i.e., sustaining electron flow through PSII and/or an additional electron sink must had been activated in E+ plants.

We have shown a threefold increase in the activity of the NADPH-MDH enzyme in *Epichloë* colonized plants. Some of this malate can be oxidized in the mitochondrion to synthesize ATP. It can also be oxidized back to oxaloacetate in the cytosol, generating NADH (Scheibe 2004). In the mitochondria malate generated by photosynthetic activity can be oxidize, providing ATP to support UDPG formation for sugar synthesis and allows the oxidation of glycine generated during photorespiration (Scheibe 2004). It cannot be ruled out that the malate synthesized by the NADPH-MDH serves as an additional energy resource for the plant due to its elevated energy demand. The endosymbiotic fungi present in the plant apoplast could in this case use malate or a product of its metabolism as a source of energy.

The results of our study shed a new light on mechanisms allowing plants to cope with the withdrawal of a significant fraction of its energy resources by endophytic fungi, allowing it at the same time to sustain improved growth. We also suggest that upon endophyte colonization, its host *D. glomerata* undergoes changes in its photosynthetic apparatus, leading to increased light harvesting and photosynthesis efficiency. This may be to support its fungal partners’ energy demands. According to the literature, *D. glomerata* benefits from the fungal antiherbivory substances (Yuan et al. 2009; Czarnoleski et al. 2012). Thus, it cannot be ruled out that this additional protection allows it to focus its own resources on carbon assimilation. The assimilated carbon may be transported, as proposed here via malate, from the chloroplast to the apoplast, where the fungi reside. This, however, needs further confirmation. Further investigations will allow us to gain a better understanding of communication and resource exchange between symbiotic plants and fungi.

### *Author contribution*

Rozpądek P—MS preparation, chlorophyll a fluorescence, idea; Wężowicz K—western blotting; Ważny R—western blotting, plant cultivation; Nosek M—chlorophyll concentration, NADPH-ME activity; Tokarz K—gas exchange measurements; Lembicz M—plant and fungi preparation, idea; Miszalski Z—MS preparation; Turnau K—MS preparation, endophyte presence verification.

## Electronic supplementary material

Supplementary material 1 (PPTX 211 kb)
