# Supplementary material for: The fungal endophyte Epichloë typhina improves photosynthesis efficiency of its host orchard grass (Dactylis glomerata)
Source: Planta. 2015 Jun 10;242(4):1025–35. doi: 10.1007/s00425-015-2337-x (PMC4560772; doi:10.1007/s00425-015-2337-x)
Supplement: Supplementary file 1 — Supplementary material 1 (PPTX 211 kb) [file 425_2015_2337_MOESM1_ESM.pptx]

## Slide 1
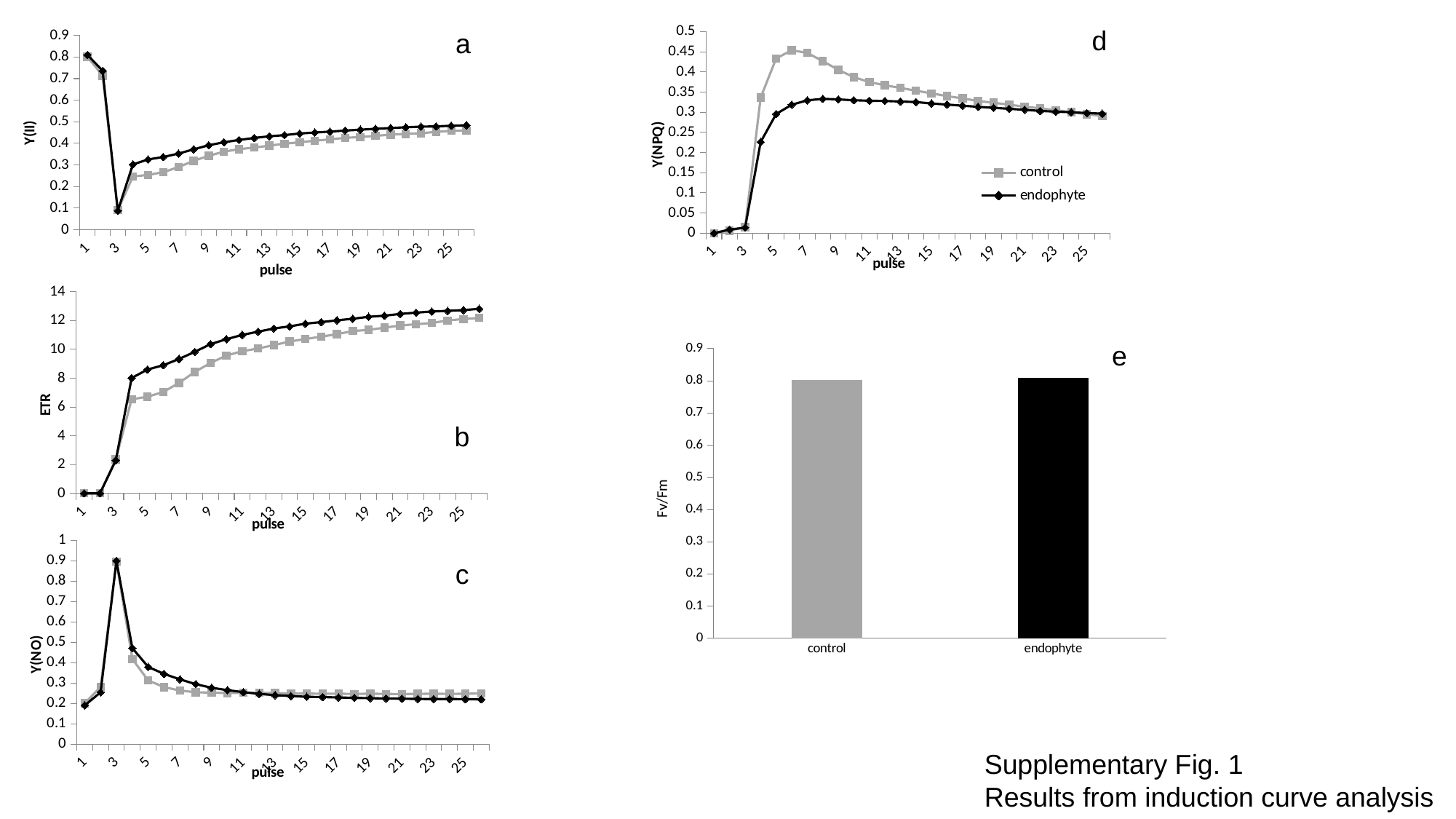

### Chart
| Category | | |
|---|---|---|a
### Chart
| Category | | |
|---|---|---|
### Chart
| Category | | |
|---|---|---|
### Chart
| Category | |
|---|---|
| control | 0.8013750000000002 |
| endophyte | 0.8102222222222223 |
### Chart
| Category | | |
|---|---|---|Supplementary Fig. 1
Results from induction curve analysis
